# Editorial: Ramadan intermittent fasting model as a catalyst for healthy aging and disease mitigation

**DOI:** 10.3389/fnut.2026.1890638

**Published:** 2026-07-09

**Authors:** MoezAlIslam E. Faris, Faiza Kalam, Meghit Boumediene Khaled, Ahmed S. BaHammam

**Affiliations:** 1 Department of Clinical Nutrition and Dietetics, Faculty of Allied Medical Sciences, Applied Science Private University, Amman, Jordan; 2 The Ohio State University Comprehensive Cancer Center, Columbus, OH, United States; 3 Division of Cancer Prevention and Control, Department of Internal Medicine, College of Medicine, The Ohio State University, The Ohio State University, Columbus, OH, United States; 4 Department of Biology, Faculty of Life and Natural Sciences, Djillali Liabes University, Lab-NuPABS, Sidi Bel Abbes, Algeria; 5 The University Sleep Disorders Center, Department of Medicine, College of Medicine, King Saud University, Riyadh, Saudi Arabia

**Keywords:** autophagy, cardiometabolic health, chrono-nutrition, inflammaging, Ramadan diurnal intermittent fasting

The growing interest in Ramadan intermittent fasting (RIF) has stimulated research into its effect on human physiology as a unique model for time-restricted eating (1). One emerging area of interest is the role of RIF in improving metabolic health and supporting healthy aging. By shifting the body's primary fuel source and triggering specific cellular signaling pathways, RIF offers a naturalistic framework to examine how periodic metabolic switching modulates glucose homeostasis (2), lipid metabolism (3), and systemic inflammation (3–5).

Beyond its immediate metabolic regulation, RIF has demonstrated promising effects on hallmarks of biological aging, including oxidative stress biomarkers (6) and autophagy activation (7–9). These mechanisms may attenuate the progression of chronic diseases and age-related physiological decline (10). Accumulating evidence suggests that RIF modulates gene expression implicated in longevity pathways and cellular stress response (5, 11, 12), thereby offering novel insights into how this culturally embedded practice can be leveraged for biogerontological benefit.

## Key themes in this Research Topic

This Research Topic, entitled “*Ramadan Intermittent Fasting Model as a Catalyst for Healthy Aging and Disease Mitigation*,” encompasses a diverse range of investigations that bridge the gap between traditional fasting and contemporary health sciences, highlighting the RIF model as a non-pharmacological strategy for disease mitigation. The five peer-reviewed articles collectively broaden current understanding of how fasting relates to immune aging and metabolic adaptation, with attention to vulnerable populations.

A central theme in this Research Topic is the modulation of the gut microbiome and immune senescence. Shahbazi et al. synthesized current evidence demonstrating how RIF promotes microbial richness and shifts in key taxa—such as *Akkermansia muciniphila*—which support intestinal barrier function and metabolic health. Complementing this work, Alkawamleh et al. investigated the potential of IF to counteract immunosenescence and inflammaging. Their review highlights how fasting-induced molecular pathways, including AMPK-SIRT1 and ketone signaling, may confer neuroprotective effects against neuroinflammation and attenuate the onset of frailty in aging populations.

The interaction between behavioral adaptation and metabolic outcomes represents another critical area of investigation. Selen provides prospective evidence on hedonic hunger, suggesting that although the “power of food” rises at the start of Ramadan, scores return to pre-fasting levels by the final week. This transient response, alongside modest reductions in body weight observed over the month, suggests that adaptation to RIF may attenuate food-related cravings. However, longer studies are needed to confirm whether such adaptation persists beyond Ramadan.

Furthermore, this Research Topic examines the combined effects of fasting with other interventions and the safety of this combination in clinical populations. Guo et al. use an umbrella systematic review to report that combining IF with intermittent hypoxia training can improve memory and attention in adults with obesity. However, heterogeneity across included studies was high. Finally, Alamri et al. examined fasting in a vulnerable group, reporting that medical education and individualized care help pregnant women with diabetes observe Ramadan more safely.

## Future directions in Ramadan and health research

While the studies in this Research Topic offer significant advancements, several key areas require further investigation to solidify RIF's place in clinical biogerontology. Long-term follow-up studies are needed to evaluate whether the microbial and immunological adaptations triggered during Ramadan persist beyond the fasting month and translate into measurable health benefits. Given interindividual variability in metabolic responses, future work should use multi-omics approaches, including metabolomics and proteomics, to inform personalized fasting protocols tailored to a person's genetic background and clinical status. Defining the unique features of RIF, which involves total fluid abstinence, compared with other intermittent fasting models, will help refine public health recommendations across diverse populations. Finally, well-designed trials in patient populations with chronic illnesses are needed to inform international guidelines and to ensure that fasting recommendations remain evidence-based and culturally appropriate.

## Conclusion

RIF serves as a useful ecological model for understanding the broader implications of dietary timing on human longevity. This Research Topic presents evidence that RIF represents a structured intervention with physiological effects that go beyond changes in body weight. The findings collected here help refine fasting strategies and inform ongoing work on health and aging in fasting populations.
